# Supplementary material for: Screening for consistency and contamination within and between bottles of 29 herbal supplements
Source: PLoS One. 2021 Nov 23;16(11):e0260463. doi: 10.1371/journal.pone.0260463 (PMC8610273; doi:10.1371/journal.pone.0260463)
Supplement: S1 Table — P-values are based on ANOVA with an α level of 0.05. (PDF) [file pone.0260463.s001.pdf]

S1 Table. P-values of the analysis of herbal supplements from two or more suppliers. P-values are based on ANOVA with an α level of 0.05.

		Water Extraction			Methanolic Extraction		
**Supplement**	**Suppliers**	**Antioxidant**	**Phenolic**	**Flavonoid**	**Antioxidant**	**Phenolic**	**Flavonoid**
Astragalus	2	<0.001	0.158	0.128	0.231	<0.001	0.002
Echinacea	2	<0.001	<0.001	<0.001	<0.001	0.567	<0.001
Echinacea Goldenseal	2	<0.001	<0.001	0.026	<0.001	<0.001	<0.001
Ginger Root	2	0.001	NS	<0.001	<0.001	<0.001	<0.001
Ginseng	2	<0.001	<0.001	0.002	0.005	<0.001	<0.001
Rhodiola	2	<0.001	0.004	<0.001	<0.001	<0.001	<0.001
St. John's Wort	3	0.26	<0.001	<0.001	<0.001	<0.001	<0.001
Turmeric	3	<0.001	<0.001	<0.001	<0.001	<0.001	<0.001
Valerian Root	3	<0.001	<0.001	0.036	<0.001	<0.001	<0.001
