# Supplementary material for: Screening for consistency and contamination within and between bottles of 29 herbal supplements
Source: PLoS One. 2021 Nov 23;16(11):e0260463. doi: 10.1371/journal.pone.0260463 (PMC8610273; doi:10.1371/journal.pone.0260463)
Supplement: S2 Table — P-values are based on ANOVA with an α level of 0.05. (PDF) [file pone.0260463.s002.pdf]

S2 Table. P-values of the analysis of bottles within a supplier for supplement. P-values are based on ANOVA with an α level of 0.05.

		Water Extraction			Methanol Extraction		
**Supplier**	**Supplement**	**A***	**P**	**F**	**A**	**P**	**F**
Nature's Way	Aloe	0.013	0.367	0.728	0.001	0.295	0.088
Nature's Way	Astragalus	0.002	0.221	0.083	<0.001	0.009	<0.001
NOW	Astragalus	<0.001	0.01	0.152	<0.001	<0.001	0.173
NOW	Biotin	<0.001	0.441	0.017	<0.001	<0.001	<0.001
Nature's Way	Cranberry	0.343	<0.001	0.777	<0.001	0.007	<0.001
Spring Valley	Echinacea	<0.001	0.065	<0.001	0.65	<0.001	<0.001
Sundown Naturals	Echinacea	<0.001	<0.001	<0.001	0.02	0.454	0.028
Nature's Way	Echinacea Goldenseal	0.878	<0.001	0.098	<0.001	<0.001	0.603
Spring Valley	Echinacea Goldenseal	0.001	0.686	0.081	<0.001	<0.001	0.046
NOW	Ginger	0.003	0.251	0.086	<0.001	0.488	0.002
Spring Valley	Ginger	0.596	0.248	0.109	0.071	0.006	0.017
Sundown Naturals	Ginseng Xtra	<0.001	<0.001	0.001	<0.001	<0.001	<0.001
Spring Valley	Korean Panax Ginseng	<0.001	<0.001	<0.001	<0.001	<0.001	0.001
Nature's Way	Red Raspberry Leaf	<0.001	<0.001	0.071	<0.001	0.393	<0.001
Nature's Way	Reishi	0.537	<0.001	0.106	<0.001	0.385	0.005
Nature's Way	Rhodiola	0.836	0.011	0.156	0.002	0.004	0.025
NOW	Rhodiola	0.001	0.039	<0.001	<0.001	<0.001	<0.001
Nature's Way	Silent Night	<0.001	<0.001	<0.001	0.172	<0.001	<0.001
Nature's Way	St. John's Wort	<0.001	0.66	0.347	<0.001	0.541	0.373

Spring Valley	St. John's Wort	<0.001	0.375	0.813	<0.001	0.034	<0.001
Sundown Naturals	St. John's Wort	<0.001	0.005	<0.001	<0.001	0.002	0.003
Sundown Naturals	Stress formula	<0.001	<0.001	0.202	0.0034	0.053	<0.001
Nature's Way	Turmeric	0.009	0.75	1.00	0.034	0.521	0.015
Spring Valley	Turmeric	0.103	0.463	0.001	<0.001	<0.001	<0.001
Sundown Naturals	Turmeric	0.288	0.001	0.005	0.001	0.76	0.30
Nature's Way	Valerian Root	<0.001	0.001	0.03	0.032	0.321	0.111
Spring Valley	Valerian Root	0.522	<0.001	<0.001	<0.001	0.006	<0.001
Sundown Naturals	Valerian Root	<0.001	0.078	0.039	0.076	0.01	0.502
Nature's Way	Yarrow	0.008	0.743	0.301	0.005	0.847	<0.001
