# Supplementary material for: Screening for consistency and contamination within and between bottles of 29 herbal supplements
Source: PLoS One. 2021 Nov 23;16(11):e0260463. doi: 10.1371/journal.pone.0260463 (PMC8610273; doi:10.1371/journal.pone.0260463)
Supplement: S3 Table — Antioxidant capacity, phenolic concentrations, and flavonoid concentrations were measured from both hot water and methanolic extractions of 10 pills from each of two bottles per supplement per supplier. Results of the Rank Runs test are also included with p-value of < 0.05. indicating non-randomness of distribution among the results. (PDF) [file pone.0260463.s003.pdf]

S3 Table. Range of coefficient of variations (CV) from 58 bottles of over the counter herbal supplements. Antioxidant capacity, phenolic concentrations, and flavonoid concentrations were measured from both hot water and methanolic extractions of 10 pills from each of two bottles per supplement per supplier. Results of the Rank Runs test are also included with p-value of < 0.05 . indicating non-randomness of distribution among the results.

		Water Extraction			Methanolic Extraction		
**Supplement**	**Suppliers**	**Antioxidant**	**Phenolic**	**Flavonoid**	**Antioxidant**	**Phenolic**	**Flavonoid**
Aloe	1	6.29-34.27	4.23-31.93	2.67-55.40	9.87-11.03	21.01-22.60	13.85-18.89
Astragalus	2	9.34-48.62	13.39-38.60	7.23-122.55	6.88-66.72	7.85-20.67	9.24-44.39
Biotin	1	22.23-89.13	18.81-38.71	0-120	4.13-161.93	25.24-30.10	0-61.70
Cranberry	1	9.79-44.42	8.39-22.15	10.65-68.28	14.22-20.64	26.90-39.01	34.59-43.47
Echinacea	2	7.89-59.95	14.16-33.17	8.21-25.39	6.49-19.41	15.99-187.57	8.90-61.54
Echinacea Goldenseal	2	9.07-48.99	9.59-33.33	9.27-34.89	2.53-20.71	12.89-47.92	5.59-109.15
Ginger Root	2	8.89-68.47	13.08-40.34	9.05-15.49	3.08-27.77	7.44-33.48	6.07- 300
Ginseng	2	14.36-21.23	26.78-39.37	0-9.05	22.53-27.77	19.56-24.74	13.50-300
Raspberry	1	15.12-19.44	5.26-6.80	4.18-8.49	4.81-5.46	11.82-23.30	4.43-5.95
Reishi	1	9.08-66.15	15.65-26.32	3.33-65.56	4.10-49.90	21.29-37.46	16.58-85.03
Rhodiola	2	14.92-28.32	11.11-61.57	8.67-30.61	18.20-76.74	14.49-38.45	14.84-38.66
Silent Night	1	10.86-23.26	6.76-23.57	12.69-30.14	10.54-49.61	6.99-17.10	9.37-53.23
St. John's Wort	3	6.10-29.28	9.14-36.18	11.15-26.44	5.17-33.89	9.00-22.51	8.03-14.67
Stress Formula	1	13.37-18.67	14.29-17.95	10.36-17.95	2.81-9.91	21.63-84.20	8.70-19.89
Turmeric	3	9.81-81.06	18.38-40.59	0-71.04	9.99-28.72	13.10-37.41	8.85-34.31
Valerian Root	3	3.45-256.21	7.56-31.90	10.20-39.06	5.23-36.01	16.68-75.11	12.90-54.16
Yarrow	1	24.06-34.58	38.81-39.45	24.53-28.12	5.09-12.22	21.86-23.18	7.82-21.63
	Rank Runs Test (p-value)	0.114	0.114	0.993	0.993	0.008	0.002
