# Supplementary material for: Screening for consistency and contamination within and between bottles of 29 herbal supplements
Source: PLoS One. 2021 Nov 23;16(11):e0260463. doi: 10.1371/journal.pone.0260463 (PMC8610273; doi:10.1371/journal.pone.0260463)
Supplement: S4 Table — T-tests indicated a change in antioxidant capacity, phenolic and flavonoid concentration of -25% from the original test. (PDF) [file pone.0260463.s004.pdf]

S4 Table. P-value from a 1 tailed t-test. T-tests indicated a change in antioxidant capacity, phenolic and flavonoid concentration of -25% from the original test.

			Water Extractions			Methanol Extractions		
**Supplier**	**Supplement**	**Bottle**	**A**	**P**	**F**	**A**	**P**	**F**
Spring Valley	Echinacea	1	0.995	<0.001	0.992	0.151	0.987	1.00
Spring Valley	Echinacea	2	<0.001	0.919	0.008	0.534	.987	0.992
Sundown Naturals	Echinacea	1	0.989	0.993	<0.001	<0.001	0.912	0.839
Sundown Naturals	Echinacea	2	<0.001	0.916	<0.001	0.021	0.84	0.207
Nature's Way	Echinacea Goldenseal	1	<0.001	0.993	<0.001	<0.001	<0.001	1.00
Nature's Way	Echinacea Goldenseal	2	0.001	0.601	<0.001	<0.001	0.81	1.00
Spring Valley	Echinacea Goldenseal	1	0.26	0.892	<0.001	<0.001	0.002	1.00
Spring Valley	Echinacea Goldenseal	2	0.999	0.882	<0.001	0.002	0.996	0.648
Spring Valley	Turmeric	1	0.508	0.903	<0.001	<0.001	1.00	0.003
Spring Valley	Turmeric	2	0.003	0.896	<0.001	<0.001	0.296	<0.001
Sundown Naturals	Turmeric	1	<0.001	0.73	<0.001	<0.001	0.006	<0.001
Sundown Naturals	Turmeric	2	<0.001	0.003	<0.001	<0.001	0.94	0.001
Nature's Way	Valerian	1	<0.001	0.972	<0.001	<0.001	0.998	1.00
Nature's Way	Valerian	2	<0.001	0.992	0.002	<0.001	0.999	0.855
Spring Valley	Valerian	1	1.00	0.956	<0.001	<0.001	0.998	0.078
Spring Valley	Valerian	2	0.023	0.348	0.019	<0.001	0.998	0.006
Sundown Naturals	Valerian	1	<0.001	0.474	<0.001	<0.001	0.419	0.938
Sundown Naturals	Valerian	2	0.005	0.707	<0.001	<0.001	0.066	0.938
