# Supplementary material for: Screening for consistency and contamination within and between bottles of 29 herbal supplements
Source: PLoS One. 2021 Nov 23;16(11):e0260463. doi: 10.1371/journal.pone.0260463 (PMC8610273; doi:10.1371/journal.pone.0260463)
Supplement: Attachment — Submitted filename: Submission Report PONE D PONE-D-20-06630R2.pdf [file pone.0260463.s007.pdf]

UNIVERSITY OF NAIROBI
COLLEGE OF AGRICULTURE AND VETERINARY SCIENCES
DEPARTMENT OF FOOD SCIENCE, NUTRITION AND TECHNOLOGY
P.O. BOX 29053-00625, KANGEMI, NAIROBI

TEL: 630172, 631353/50/48- EXTENSION: 27093: E-mail: ckunyanga@uonbi.ac.ke

7th January, 2020

REF: ACADEMIC EDITORS COMMENTS FOR MANUSCRIPT PONE-D-20-06630R2

Title: Screening for consistency and contamination within and between bottles of 29 herbal supplements.

The manuscript has been thoroughly revised based on the reviewer's comments. The authors have adhered to the journal guidelines in preparation and submission of the revised manuscript. All the relevant documentation to support the manuscript including ethic consideration clearance have been provided by the authors.

The manuscript can now be considered for publication as submitted.

Yours Sincerely,

Dr. Catherine Kunyanga (PhD)
Department of Food Science, Nutrition & Technology
