# Supplementary material for: Screening for consistency and contamination within and between bottles of 29 herbal supplements
Source: PLoS One. 2021 Nov 23;16(11):e0260463. doi: 10.1371/journal.pone.0260463 (PMC8610273; doi:10.1371/journal.pone.0260463)
Supplement: Attachment — Submitted filename: Academic editor report PONE D 20 06630.pdf [file pone.0260463.s009.pdf]

UNIVERSITY OF NAIROBI
COLLEGE OF AGRICULTURE AND VETERINARY SCIENCES
DEPARTMENT OF FOOD SCIENCE, NUTRITION AND TECHNOLOGY
P.O. BOX 29053-00625, KANGEMI, NAIROBI

TEL: 630172, 631353/50/48- EXTENSION: 27093: E-mail: ckunyanga@uonbi.ac.ke

16th February, 2021

REF: ACADEMIC EDITOR'S COMMENTS FOR MANUSCRIPT PONE-D-20-06630

Title: Screening for consistency and contamination within and between bottles of 29 herbal supplements.

The manuscript has been adequately revised to incorporate the comments by reviewers and this improves the quality. The following were checked in the revised version.

- The authors have revised the statistical analysis and presented the results in the manuscript.
- The material and method sections have been revised to incorporate standard protocols. Subsections for sample collection, laboratory analysis, sample extraction, antioxidant, stability test, metals test, physical contamination and fungi test were added.
- The discussion section and subsections has been added and revised.
- Line 21-23, the quantitative value/data (mean±sd) for antioxidant activity, phenolic concentrations, and flavonoid concentrations have been stated.
- Line 15. Bracket (FDA) after the Food and Drug Administration (FDA), were added.
- Line 33 Key Words, were revised according to PLOS authors guide.
- Line 99. Please make subsections for Materials and Methods.
- Personal pronouns (we) in the manuscript were modified in Line 101, 102, 104 122, 141, 152, 158, 169, 180, 201, 204, 219, 224, 241, 245, 282, 285.
- Title of Table 1 has been revised.

- Line 113 Source/reference for extraction, has been added (Cai, Y., Luo, Q., Sun, M., Corke, H., 2004. Antioxidant activity and phenolic compounds of 112 traditional Chinese medicinal plants associated with anticancer. Life Sci. 74, 2157-2184).
- Line 118/113 Source for the extraction protocol were added and ecplanation of supplement source added.
- Line 119. FRAP is written in full (Ferric reducing antioxidant power) and FRAP in the bracket.
- Line 119. Explanation for choice of one assay has been provided ("Recognizing that there are multiple methods of measuring antioxidants, phenolics and flavonoids it was decided that one type of assay for each should be conducted to more quickly determine if there is justification for further investigation into the purity and consistency of the supplements.").
- Line 130, Explanation for source for the physical contamination test has been provided.
- Line 139 and 140 Source was added (Singh P, Srivastava B, Kumar A, Dubey NK. Contamination of raw materials of some herbal drugs and recommendation of cinnamomum camphora oil as herbal fungitoxicant. Micro Ecol. 2008; 56: 555-560).
- Line 148,149, the alpha level for the degree of significance has been revised.
- Line 170. Table 2 - data has been split between 2 tables. One based on the means and SE for each bottle of each supplement and supplier. The other is based on the overall means for each supplier of each supplement (now tables 2 and 3)
- Line 170. title of Table 2, and footnote at the bottom of the Table has been revised.
- Line 188 title of Table 3 has been revised.
- Table 3 has been changed to show the CV for all values, but we believe that the CV is valuable in evaluating supplements with a wide range of values.
- Table 3. superscript letters have been added to show the degree of significance.

- Line 209 Table 4 title has been modified
- Lines 83-86: Links to FDA sources describing the three 2015 recalls reported are now included in the reference section under reference 28.
- Materials and Methods section – The following statement has been added at the end of the first paragraph of the materials and methods. – " All of the extractions and tests, with the exception of the supplement stability tests were conducted from October 2016 through April 2017. The supplement stability tests were conducted in January and February 2019."
- Lines 184-85: The wording has been changed to show that differences reported among bottles indicate a significant difference.
- Lines 237-38 & 297-306: The wording here has been changed to the following, which hopefully removes a causal connection – "Although the amount of copper within the supplement is most likely not problematic for the majority of the population, it could unintentionally lead to dangerous copper levels in individuals who already have higher than normal copper uptake or susceptible populations"
- Lines 244-48: The following was added "The bottle was purchased at Walmart in Maryland at the same time as the purchase of the replicate bottle."

The above revisions have been duly noted as addressed by the authors in the revised submission.

Yours Sincerely,

Dr. Catherine Kunyanga (PhD)
Department of Food Science, Nutrition & Technology
