# Supplementary material for: Screening for consistency and contamination within and between bottles of 29 herbal supplements
Source: PLoS One. 2021 Nov 23;16(11):e0260463. doi: 10.1371/journal.pone.0260463 (PMC8610273; doi:10.1371/journal.pone.0260463)
Supplement: Attachment — Submitted filename: Response to Reviewer comments_Plos R2.docx [file pone.0260463.s006.docx]

Response to Reviewer comments

- We note that line 68 is not supported by citation to a reference, but this could be because it may be intended to set up the reference in line 76. Please confirm or revise as appropriate.  As currently phrased, it could reads like manufacturers intentionally contaminated supplements. Is this what the authors intended to say?  If not, perhaps revise like this: “Contamination may be due to growth, processing or storage conditions or may be intentional addition of unlisted ingredients.” – The wording has been changed as per your suggestion. The sentence was to provide a set up for the rest of the paragraph.
- Lns 83-86:  Please provide links to FDA sources describing the three 2015 recalls reported.  – The archived links are now included in the reference section under reference 28.
- In the Materials and Methods section, please specify exactly when the testing was done. – The following statement was added at the end of the first paragraph of the materials and methods. – “ All of the extractions and tests, with the exception of the supplement stability tests were conducted from October 2016 through April 2017. The supplement stability tests were conducted in January and February 2019.”
- Lns 184-85:  Please clarify whether any differences reported were statistically significant. – The wording has been changed to show that differences reported among bottles indicate a significant difference.
- Lns 237-38 & 297-306: Is the suggestion that ingesting copper levels detected in Spring Valley’s St. John’s Wort over time result in copper toxicity bad enough to cause Alzheimer’s, Parkinson’s or cognitive decline? The current phrasing seems to imply this is the case, even when taken a “correct dosage” (at least with respect to cognitive decline). This should be substantiated. – The wording here has been changed to the following, which hopefully removes a causalconnection – “Although the amount of copper within the supplement is most likely not problematic for the majority of the population, it could unintentionally lead to dangerous copper levels in individuals who already have higher than normal copper uptake or susceptible populations”
- Lns 244-48:  Please indicate where the bottle described in these lines was purchased to avoid any argument that the bottle tested was not a genuine bottle but a counterfeit.   – The following was added “The bottle was purchased at Walmart in Maryland at the same time as the purchase of the replicate bottle.”

Hopefully, the authors will consider all the comments to maintain the quality of the manuscript for the readership of the PLOS community.
As a reviewer, I am still sticking to my previous recommendation. Additional data is necessary to consider this paper as a full research manuscript, and need to improve and thoroughly discuss the tables and figures.

2. Line 21-23, Please state the quantitative value/data (mean±sd) for antioxidant
activity, phenolic concentrations, and flavonoid concentrations.
– Included one the range of CVs as an example, but it would be impractical to include the quantitative data for all of the supplements in a meaningful way within the abstract of the manuscript. In this case the CV is ideal as it gives an idea of variability that is standardized across values that are different by orders of magnitude.
My request is to include the mean±sd of antioxidant activity, phenolic concentrations, and flavonoid concentrations in the abstract section. The authors do not address this. – The coefficient of variation is calculated as a ratio of the mean to the standard deviation. It is specifically used in cases where the means are so different in a scale of magnitude that comparing one mean to the other would not be feasible. To include any kind of single overall mean ± std for antioxidants, phenolics and flavonoids would be useless. The values between supplements are so different that any sort of composite mean would not be representative of any of the values presented within the tables in the manuscript. Because the coefficient of variation is a measure of the relative standard deviation it is a much more efficient way to encapsulate what the reviewer wants in a very concise format that is also meaningful.
The description of statistical variation needs to be based on mean±sd, and the superscript should be stated for each treatment, which is based on the treatment variation. (This is also seen in the new document, line number 163 page 10). – I’m not sure how the reviewer expects for this to be included within the abstract since the values for each of these are so vastly different and that any composite mean would be meaningless. See comment above.

10.Line 119. For antioxidant in vitro test, at least two assays needed; in addition, better also to look the Effective concentration (EC50). These make the antioxidant activity test/data to the standard and trustful.
– The reason for choosing only one type of assay for each type is included at the beginning of the discussion. “Recognizing that there are multiple methods of measuring antioxidants, phenolics and flavonoids it was decided that one type of assay for each should be conducted to more quickly determine if there
is justification for further investigation into the purity and consistency of the
supplements.”
Phenol and flavonoids are phytochemicals, which are considered as antioxidant content. My request is for antioxidant activity, like FRAP. In line with this calculating EC50 increases the readability of the manuscript, and also makes the data trustful. – The samples and supplements that we tested have long since passed the time where an additional antioxidant test, such as the EC50 could be performed on these bottles. However, in addition to the statement included in the first revision this statement has also been added to the discussion to indicate that further testing is justified and needed – “and that further testing involving different assays for antioxidant capacity and specific phenolics and flavonoids is justified for these supplements”

13. Line 148,149, please state the alpha level for the degree of significance.
- Done
The authors said done for this comment; however, the alpha level for the degree of significance is not in place. – The alpha level for each test is listed in the section under statistical analysis which is copied here. The alpha level was also included in the footnotes of the tables where appropriate. “**Statistical Analysis.** For each bottle of each supplement, the means and standard error were calculated for the water, methanol, and nitric acid extractions. In addition, the coefficient of variation (CV) was calculated for the water and methanol extractions. Comparisons among suppliers and bottles from the same supplier were done using the general linear model in the FitModel platform of JMP with an **α level of 0.05** [38]. A runs test was also conducted to test for randomness of rankings among bottles, suppliers and supplements using Minitab® Statistical Software with an **α level of 0.05** [39]. A significant runs test means suppliers and/or supplements clump together. To determine if there had been a change in the average values for the antioxidant, phenolic and flavonoid assays after two years of supplement storage the data was analyzed using a one-tailed t-test with an **α level of 0.05** and a test value of < -25% change using Minitab® Statistical Software [39].

14. Line 170. Table 2 needs a general modification. This table should be with the numerical value (mean±se for each supplement and parameters). The mean values should be also differentiated using superscript letters if significant differences are found.
– This data is now split between 2 tables. One based on the means and SE for each bottle of each supplement and supplier. The other is based on the overall means for each supplier of each supplement (now tables 2 and 3).
The superscript stated in Table 2 and 3 are not correct, and is not statistical accepted. Some of the values do not have also the superscripts. – I’m not really sure why the reviewer states that the superscripts are not correct. I have made the following modifications to hopefully address the reviewers concerns. In table 2 a ^ was placed next to values from supplements where there is only one supplier so that comparisons among suppliers is not possible. In addition, I have placed a superscript letter next to all values, even those that are not significantly different. In addition our data set with statistical analysis has been reorganized and updated in the open source data link to make it easier to see all of the analysis. <https://doi.org/10.7910/DVN/TRLTI3>

17.Table 3. Better to describe as mean±sd, please.
– The table has been changed to show the CV for all values, but we believe that the CV is valuable in evaluating supplements with a wide range of values. Therefore we are including an expanded CV table in addition to the means and standard errors with accompanying superscripts in two new tables. See 15 above.
To my knowledge, CV does not indicate a statistical variation between treatments. For a reputable journal like PLOS ONE, a description of treatments using a statistical variation is important for the readers, and maintain the quality of the journal. – The purpose of the coefficient of variation is to allow for a snapshot of how much variation there is around a mean without reference directly to the mean and it’s attendant units. It is also useful in examining variation in tests that do not have similar means as it is a measure of the relative standard deviation (RSD)

- 18. Table 3. needs to have superscript letters to show the degree of significance. –Done.
  This is not properly done. – The table has been modified to include superscript letters for all values, even those without significant differences between bottles. Initially we had not included superscripts for the insignificant values to reduce the amount of clutter within the table.

  19. Table 3. An antioxidant is a broad term that does not described as a single
  parameter. It needs to be specific. In your case, FRAP. Please know that a single in vitro parameter is not to the standard to support the antioxidant activity of your samples. Please refer to the comment give in Line 119. – See response to comment 11 above.
  This is not addressed. – To address this FRAP is included in all table titles in association with antioxidant capacity, but within the body of the tables we use only ‘A’ to symbolize this category in keeping with the single letter symbols ‘P’ and ‘F’ for phenolics and flavonoids.

  20.Line 209 Table 4, please modify the title of the Table, state mean±sd with significant superscript letters. The range value can be indicated in your discussion.
  – The title has been modified.
  The title is modified; however, it is necessary to address the other requests. – Table 4 in the old manuscript is now table 6 in revision 1. For the metals we were simply testing for the presence versus absence of the metals within the supplements and did not conduct an ANOVA on the data.
