# Supplementary material for: Screening for consistency and contamination within and between bottles of 29 herbal supplements
Source: PLoS One. 2021 Nov 23;16(11):e0260463. doi: 10.1371/journal.pone.0260463 (PMC8610273; doi:10.1371/journal.pone.0260463)
Supplement: Attachment — Submitted filename: Response to Reviewer Comments_Plos R3.docx [file pone.0260463.s008.docx]

**Response to Reviewers _R3**

1. Supplement stability tests were in some cases performed after the expiration date listed, but in others before. Please include some text discussing this as a limitation, and consider whether you could include subgroup analysis separating out the supplements that had expired from those that had not in the stability tests.***** **There was no consistent pattern displayed by bottles within their use by date versus those that had expired. When there was a significant difference between the supplements that had expired and those that had not in all but one case the expired supplement had more stability than the non-expired supplement (data not shown). Valerian root that had expired had a more significant decrease in flavonoid content in the water soluble fraction; however, all bottles saw a decrease of more than 25% (data not shown).*. In addition we added ^ in the table next to the supplements that had expired and indicated that within the footnote.***

2. Please clarify whether the supplements were stored according to the package instructions, and were they unopened before testing? Storage conditions are currently described as having “mimicked storage conditions in a home”, but there is no formal description of these conditions (in terms of temperature, humidity etc.) ***The following was added to the methods section .“All bottles were sealed and stored away from light at room temperature (between 20 and 25° C) until initial analysis. After opening, the bottles were stored with the lids tightly closed under the same temperature and light conditions. The humidity within the lab ranged between 55 and 65%. In addition, each bottle was stored with the included silica pack to reduce moisture accumulation within the bottles.”**

3. Please describe where all of the tested supplements were purchased e.g. online or a local store (please provide names). - ***The following sentence was placed in the ‘Supplement Selection’ section. “All Spring Valley supplements were purchased in person at Walmart in Cockeysville, Maryland as were the Sundown Naturals Turmeric and Stress Formula. All other supplements were purchased online through the Amazon Marketplace.”**

4. Regarding the recalls (lines 83-88), in reading the archived FDA alerts, it seems that the companies themselves voluntarily recalled the products, not the FDA. We would recommend removing this sentence and the next in their entirety – they are not necessary to support the claims in this paper. - ***These lines have been removed and the references renumbered.**
