# Supplementary material for: Screening for consistency and contamination within and between bottles of 29 herbal supplements
Source: PLoS One. 2021 Nov 23;16(11):e0260463. doi: 10.1371/journal.pone.0260463 (PMC8610273; doi:10.1371/journal.pone.0260463)
Supplement: Attachment — Submitted filename: Response to reviewers Plos One_R4.docx [file pone.0260463.s010.docx]

Journal Requirements:

Please review your reference list to ensure that it is complete and correct. If you have cited papers that have been retracted, please include the rationale for doing so in the manuscript text, or remove these references and replace them with relevant current references. Any changes to the reference list should be mentioned in the rebuttal letter that accompanies your revised manuscript. If you need to cite a retracted article, indicate the article’s retracted status in the References list and also include a citation and full reference for the retraction notice.

[Note: HTML markup is below. Please do not edit.]

Reviewers' comments:

Reviewer's Responses to Questions

**Comments to the Author**

1. If the authors have adequately addressed your comments raised in a previous round of review and you feel that this manuscript is now acceptable for publication, you may indicate that here to bypass the “Comments to the Author” section, enter your conflict of interest statement in the “Confidential to Editor” section, and submit your "Accept" recommendation.

Reviewer #2: All comments have been addressed

Reviewer #3: All comments have been addressed

Reviewer #4: (No Response)

Reviewer #5: (No Response)

2. Is the manuscript technically sound, and do the data support the conclusions?

The manuscript must describe a technically sound piece of scientific research with data that supports the conclusions. Experiments must have been conducted rigorously, with appropriate controls, replication, and sample sizes. The conclusions must be drawn appropriately based on the data presented.

Reviewer #2: Yes

Reviewer #3: Partly

Reviewer #4: Yes

Reviewer #5: Yes

3. Has the statistical analysis been performed appropriately and rigorously?

Reviewer #2: Yes

Reviewer #3: Yes

Reviewer #4: Yes

Reviewer #5: Yes

4. Have the authors made all data underlying the findings in their manuscript fully available?

The [PLOS Data policy](https://nam04.safelinks.protection.outlook.com/?url=http%3A%2F%2Fwww.plosone.org%2Fstatic%2Fpolicies.action%23sharing&data=04%7C01%7Cmblohm%40loyola.edu%7Cb7b5b2f598174210fd9908d9876d7794%7C30ae0a8f3cdf44fdaf34278bf639b85d%7C0%7C0%7C637689726645012903%7CUnknown%7CTWFpbGZsb3d8eyJWIjoiMC4wLjAwMDAiLCJQIjoiV2luMzIiLCJBTiI6Ik1haWwiLCJXVCI6Mn0%3D%7C1000&sdata=2G4AIR3KvpoyALpogpJBpfhoOPQXNsvToi61I4wjiQQ%3D&reserved=0) requires authors to make all data underlying the findings described in their manuscript fully available without restriction, with rare exception (please refer to the Data Availability Statement in the manuscript PDF file). The data should be provided as part of the manuscript or its supporting information, or deposited to a public repository. For example, in addition to summary statistics, the data points behind means, medians and variance measures should be available. If there are restrictions on publicly sharing data—e.g. participant privacy or use of data from a third party—those must be specified.

Reviewer #2: Yes

Reviewer #3: Yes

Reviewer #4: No

Reviewer #5: Yes

5. Is the manuscript presented in an intelligible fashion and written in standard English?

PLOS ONE does not copyedit accepted manuscripts, so the language in submitted articles must be clear, correct, and unambiguous. Any typographical or grammatical errors should be corrected at revision, so please note any specific errors here.

Reviewer #2: Yes

Reviewer #3: Yes

Reviewer #4: Yes

Reviewer #5: Yes

6. Review Comments to the Author

Please use the space provided to explain your answers to the questions above. You may also include additional comments for the author, including concerns about dual publication, research ethics, or publication ethics. (Please upload your review as an attachment if it exceeds 20,000 characters)

Reviewer #2: The authors have addressed the comments appropriately and I recommend the manuscript be accepted for publication.

Reviewer #3: 1) line 75: cancel comma before the references – Done

2)Introduction: the cited references are a little bit old. Could you find newer literature? – Four additional references from 2019 through 2021 were added to the introduction and one reference from 2020 was added to the discussion. The rest of the references we wanted to keep as they contribute to the justification for the importance of the research conducted. When the paper was first submitted almost 2 years ago at least some of the references were more recent.

3)Tables 3 and 6: what do you mean "0" is the same as not determined? – It is unclear to me what the reviewer is asking here. It is never stated in the paper that 0 is the same as not determined. When zero is listed as the mean of the value for the supplement it indicates that none of the pills tested of that supplement had any detectable amount of either the antixodant activity, phenolic or flavonoids or the metals in question.

4)in Tables with results SD is very high in some cases. – This is addressed briefly in the discussion with the following statement -- (as demonstrated by the large standard errors)
5) Please, rewrite the conclusion. - done
6) Lack of novelty.
7) Lack of the validation of the methods.

Reviewer #4:

1. Did the extraction protocol performed according to the total weight of the supplement, or according to weight of extract per gram in a supplement? No information is provided in the methods section in terms of the extract amount per weight.

- Added to the method sections- ‘All assays values were calculated and standardized based on the weight of the sample that was extracted.’
  2. Even though, morphological identification of fungal species is important and routinely used, for fungal systematics, taxonomic classification may not always perform well for species classifications. This would be a limitation which should be further addressed. – The following statement was added to the discussion – “Morphological identification of the species indicates that there were potential species of concern, which indicate targets for further research to determine if the species tentatively identified are indeed problematic.”
  3. Similarly, the gross physical contamination of the samples were screened under a light-dissecting microscope; while no information regarding the standards for the methodology (such as AOAC International, American Spice Trade Association, pharmacopeia’s, FDA’s Macroanalytical Procedures Manual etc) is provided.
- The following line was added to the methods. “The samples were scanned quickly for uniformity within a capsule and among capsules within the same bottle and between different bottles.”
- Within the results the following statement was changed “lacked gross physical contamination” and replaced with “were uniform in texture and color”

4. Also comprehensive investigation are also based on chemical techniques along with Fourier transform-infrared (FTIR) analysis; which is also a limitation and is expected to be addressed. – Included the following statement in the discussion - “An important next step is testing the supplements for the presence of and concentration of the active ingredient if present.”
5. Legal aspects in terms of chemical and microbiological quality of herbal supplements should be discussed within the respective directives.

- It wasn’t exactly clear to me what the reviewer meant by this comment. I had it checked by the university’s research counsel and they also were not certain. In order to try to address it I added the following to the conclusion –‘The data presented here strengthens the case that lack of regulation leads to products that are not standardized and have the potential to either be ineffective or to cause harm. Currently in the United States manufacturers are to ensure safety of their products, but the FDA can only legally act to remove supplements when there is proof of harm, false statements or ineffective labelling [57].’

Reviewer #5: The required revisions are made on the manuscript. It is really very important to search for the quality of herbal supplements and share the gathered results with public.

7. PLOS authors have the option to publish the peer review history of their article ([what does this mean?](https://nam04.safelinks.protection.outlook.com/?url=https%3A%2F%2Fjournals.plos.org%2Fplosone%2Fs%2Feditorial-and-peer-review-process%23loc-peer-review-history&data=04%7C01%7Cmblohm%40loyola.edu%7Cb7b5b2f598174210fd9908d9876d7794%7C30ae0a8f3cdf44fdaf34278bf639b85d%7C0%7C0%7C637689726645022899%7CUnknown%7CTWFpbGZsb3d8eyJWIjoiMC4wLjAwMDAiLCJQIjoiV2luMzIiLCJBTiI6Ik1haWwiLCJXVCI6Mn0%3D%7C1000&sdata=F9%2FUQIrf8jJCJem4Tc1%2BjfGTkU4BJPXyaIzO537IDNI%3D&reserved=0)). If published, this will include your full peer review and any attached files.


If you choose “no”, your identity will remain anonymous but your review may still be made public.


**Do you want your identity to be public for this peer review?** For information about this choice, including consent withdrawal, please see our [Privacy Policy](https://nam04.safelinks.protection.outlook.com/?url=https%3A%2F%2Fwww.plos.org%2Fprivacy-policy&data=04%7C01%7Cmblohm%40loyola.edu%7Cb7b5b2f598174210fd9908d9876d7794%7C30ae0a8f3cdf44fdaf34278bf639b85d%7C0%7C0%7C637689726645022899%7CUnknown%7CTWFpbGZsb3d8eyJWIjoiMC4wLjAwMDAiLCJQIjoiV2luMzIiLCJBTiI6Ik1haWwiLCJXVCI6Mn0%3D%7C1000&sdata=7ltQlFz4p7MOKgQbgIRA13aayGFg4Wrc4DPLa5HBZJ8%3D&reserved=0).

Reviewer #2: No

Reviewer #3: No

Reviewer #4: No

Reviewer #5: No
