# Supplementary material for: Screening for consistency and contamination within and between bottles of 29 herbal supplements
Source: PLoS One. 2021 Nov 23;16(11):e0260463. doi: 10.1371/journal.pone.0260463 (PMC8610273; doi:10.1371/journal.pone.0260463)
Supplement: Attachment — Submitted filename: PLOS One Herbals - Response to reviewer comments.docx [file pone.0260463.s005.docx]

The manuscript requires major revision to address concerns raised by reviewers.

Kindly give emphasis on the following during revisions:

1. Conduct thorough statistical analysis for data presented in the results section to make better interpretations of the findings.

2. Insert missing materials and methods section using standard protocols and give well described methodology of how the data was collected for all the parameters analyzed.

3. Include supplementary data that can add quality to the findings if you have more data pending that wasn't included in the manuscript, this increase quality of the publication.

4. Improve the discussion section to be in line with the results stated in a logical sequence.

5. Check and correct the overall quality of the manuscript based on the reviewers' comments including missing sections and editorial/grammatical errors.

Please submit your revised manuscript by 30th September, 2020. If you will need more time than this to complete your revisions, please reply to this message or contact the journal office at [plosone@plos.org](mailto:plosone@plos.org). When you're ready to submit your revision, log on to <https://www.editorialmanager.com/pone/> and select the 'Submissions Needing Revision' folder to locate your manuscript file.

Please include the following items when submitting your revised manuscript:

- A rebuttal letter that responds to each point raised by the academic editor and reviewer(s). You should upload this letter as a separate file labeled 'Response to Reviewers'.
- A marked-up copy of your manuscript that highlights changes made to the original version. You should upload this as a separate file labeled 'Revised Manuscript with Track Changes'.
- An unmarked version of your revised paper without tracked changes. You should upload this as a separate file labeled 'Manuscript'.

1. Please ensure that your manuscript meets PLOS ONE's style requirements, including those for file naming. The PLOS ONE style templates can be found at

<https://journals.plos.org/plosone/s/file?id=wjVg/PLOSOne_formatting_sample_main_body.pdf> and

<https://journals.plos.org/plosone/s/file?id=ba62/PLOSOne_formatting_sample_title_authors_affiliations.pdf>

**Comments to the Author**

1. Is the manuscript technically sound, and do the data support the conclusions?

The manuscript must describe a technically sound piece of scientific research with data that supports the conclusions. Experiments must have been conducted rigorously, with appropriate controls, replication, and sample sizes. The conclusions must be drawn appropriately based on the data presented.

Reviewer #1: No

2. Has the statistical analysis been performed appropriately and rigorously?

Reviewer #1: No

3. Have the authors made all data underlying the findings in their manuscript fully available?

The [PLOS Data policy](https://nam04.safelinks.protection.outlook.com/?url=http%3A%2F%2Fwww.plosone.org%2Fstatic%2Fpolicies.action%23sharing&data=02%7C01%7Cmblohm%40loyola.edu%7C458498f4ebe5441b24ac08d8589594f3%7C30ae0a8f3cdf44fdaf34278bf639b85d%7C0%7C1%7C637356746761252316&sdata=h9zBrdQxC73uUlnfzXJpZ0EvGC4%2FGt584pOAio2BsUU%3D&reserved=0) requires authors to make all data underlying the findings described in their manuscript fully available without restriction, with rare exception (please refer to the Data Availability Statement in the manuscript PDF file). The data should be provided as part of the manuscript or its supporting information, or deposited to a public repository. For example, in addition to summary statistics, the data points behind means, medians and variance measures should be available. If there are restrictions on publicly sharing data—e.g. participant privacy or use of data from a third party—those must be specified.

Reviewer #1: Yes

4. Is the manuscript presented in an intelligible fashion and written in standard English?

PLOS ONE does not copyedit accepted manuscripts, so the language in submitted articles must be clear, correct, and unambiguous. Any typographical or grammatical errors should be corrected at revision, so please note any specific errors here.

Reviewer #1: No – Actually the manuscript is written in standard English; although, grammatical issues related to use of first person rather than passive voice have been addressed. However, some of the reviewer’s comments were not written using correct English grammar and syntax.

5. Review Comments to the Author

1. Reviewer #1: It is a very good initiation to work on ensuring the consistency, purity, and safety of herbs relating to heavy metals and microorganisms, which have a role in adaptogens, stress and depression relief, and immune response. However, I have doubtful to consider this manuscript as a full research paper. If there is a room, PLOS can also handle as a short communication or letter. – Hopefully the addition of the complete statistical analysis will allow the paper to be considered as a full research paper.
2. Line 21-23, Please state the quantitative value/data (mean±sd) for antioxidant activity, phenolic concentrations, and flavonoid concentrations. – Included one the range of CVs as an example, but it would be impractical to include the quantitative data for all of the supplements in a meaningful way within the abstract of the manuscript. In this case the CV is ideal as it gives an idea of variability that is standardized across values that are different by orders of magnitude.
3. Line 15. Please put in bracket (FDA) after the Food and Drug Administration (FDA), as this is the 1st appearance. - Done
4. Line 33 Key Words, please make Keywords, please also check for the number of the keywords included, from PLOS authors guide. – There are already keywords included in the paper. I could not find information about key words in the authors guide
5. Line 99. Please make subsections for Materials and Methods.
   Example
   Sample collection
   Laboratory analysis
   Sample extraction
   Antioxidant
   Stability test
   Metals test
   Physical contamination
   Fungi test
   Please also make the subsections for your Discussion; based on the content of your Discussion. – Done, the number of subsections included is not as numerous as suggested in the above example as this would have impeded the flow of the paper. However, there are now subsections for the Materials and Methods, Results and Discussion
6. The authors repeatedly use personal pronouns (we) in a different part of the manuscript that needs to be changed/modified, Line 101, 102, 104 122, 141, 152, 158, 169, 180, 201, 204, 219, 224, 241, 245, 282, 285. – Done
7. Line 107, the title of Table 1 is lengthy, please revise, and if need be, put all the other descriptions at the bottom of the table in the bracket or asterisk. -Done

Line 113 please indicate the source/reference for extraction, which consists of the detailed protocol. – Done - Cai, Y., Luo, Q., Sun, M., Corke, H., 2004.  Antioxidant activity and phenolic compounds of 112 traditional Chinese medicinal plants associated with anticancer.  Life Sci. 74, 2157-2184.

8. Line 118 state the source, at the end of the sentence. – In the original manuscript only line 118 refers to an extraction protocol, while line 113 is the title for table 1. So I’ve included the source for the extraction protocol, but there was no source for what supplements were tested. These were chosen by the lab group and the subsampling was done to ensure that each pill had all 4 extractions conducted.
9. Line 119. FRAP please write Ferric reducing antioxidant power and FRAP in the bracket. - Done
10. Line 119. For antioxidant in vitro test, at least two assays needed; in addition, better also to look the Effective concentration (EC50). These make the antioxidant activity test/data to the standard and trustful. – The reason for choosing only one type of assay for each type is included at the beginning of the discussion. “Recognizing that there are multiple methods of measuring antioxidants, phenolics and flavonoids it was decided that one type of assay for each should be conducted to more quickly determine if there is justification for further investigation into the purity and consistency of the supplements.”
11. Line 130, please state a source for the physical contamination test. – There is no source for this. We just examined the samples under the dissecting microscope to see if anything looked unusual or out of place.
12. Line 139 and 140 please state a source. – The source was added. Singh P, Srivastava B, Kumar A, Dubey NK. Contamination of raw materials of some herbal drugs and recommendation of cinnamomum camphora oil as herbal fungitoxicant. Micro Ecol. 2008; 56: 555-560.
13. Line 148,149, please state the alpha level for the degree of significance. - Done
14. Line 170. Table 2 needs a general modification.
    This table should be with the numerical value (mean±se for each supplement and parameters). The mean values should be also differentiated using superscript letters if significant differences found. – This data is now split between 2 tables. One based on the means and SE for each bottle of each supplement and supplier. The other is based on the overall means for each supplier of each supplement (now tables 2 and 3)
15. Line 170. Please state the title of Table 2, and put the others, as the footnote at the bottom of the Table. - Done
16. Line 188 please rewrite the title of Table 3. - Done
17. Table 3. Better to describe as mean±sd, please. – The table has been changed to show the CV for all values, but we believe that the CV is valuable in evaluating supplements with a wide range of values. Therefore we are including an expanded CV table in addition to the means and standard errors with accompanying superscripts in two new tables. See 15 above.
18. Table 3. needs to have superscript letters to show the degree of significance. - Done
19. Table 3. An antioxidant is a broad term that does not described as a single parameter. It needs to be specific. In your case, FRAP. Please know that a single in vitro parameter is not to the standard to support the antioxidant activity of your samples. Please refer to the comment give in Line 119. – See response to comment 11 above.
20. Line 209 Table 4, please modify the title of the Table, state mean±sd with significant superscript letters. The range value can be indicated in your discussion. – The title has been modified
21. Line 237, Our data, in conjunction with previous studies on supplement contamination, …..
    Please substitute Our data, ……
    The findings of this study/data
    Please apply these changes, wherever needed. - Done
22. Line 281 please apply the above comment (Line 237). – Done
23. Page 24 I am not clear for the question mark found in Figure 1. – I have carefully looked over the manuscript figure file and found no question mark in Figure 1.

    Thank you
    Abera Belay
